# Anti-neoplastic effects of the antipsychotic drug penfluridol in preclinical prostate cancer models

**DOI:** 10.3389/fonc.2025.1685758

**Published:** 2025-10-14

**Authors:** Arjanneke F. van de Merbel, Maaike H. van der Mark, Tilly Aalders, Martin Puhr, Niven Mehra, Jack Schalken, Geertje van der Horst, Gabri van der Pluijm

**Affiliations:** ^1^ Department of Urology, Leiden University Medical Center, Leiden, Netherlands; ^2^ Department of Urology, Radboud University Medical Center, Nijmegen, Netherlands; ^3^ Department of Urology, Medical University of Innsbruck, Innsbruck, Austria; ^4^ Department of Medical Oncology, Radboud University Medical Center, Nijmegen, Netherlands

**Keywords:** prostate cancer, preclinical models, drug repurposing, drug repositioning, penfluridol, cancer stem cells

## Abstract

**Introduction:**

The development of therapy resistance and the formation of distant metastases represent clinical unmet needs for patients with advanced prostate cancer (PCa). The use of drugs for other indications, i.e. drug repurposing, shows great promise for cancer treatment. Drug repurposing could allow new cancer treatments to be introduced relatively quickly and at lower costs. Penfluridol, an approved antipsychotic drug, has strong cytolytic effects in multiple cancers.

**Methods:**

In this study, we have investigated the potential anti-tumor effects of penfluridol in preclinical and ‘near-patient’ PCa models.

**Results:**

Penfluridol significantly reduced the viability of a panel of human PCa cells, induced apoptosis by increasing caspase-3/7 levels and decreased the number of PCa stem cells in vitro. Penfluridol reduced the viability and induced cytotoxic effects in three-dimensional cultures and in ex vivo cultured PCa tissue slices (patient-derived xenograft, freshly isolated PCa biopsies). Moreover, penfluridol significantly reduced the viability of docetaxel-resistant PCa cells and exerted synergistic effects in combination with docetaxel in docetaxel-resistant PCa.

**Discussion:**

In conclusion, penfluridol exhibited cytotoxic effects in multiple preclinical PCa models. Further research is warranted to address the translational value of our findings.

## 1 Introduction

Prostate cancer (PCa) is the second most common cancer type in men in the Western world (1). The development of castration-resistant prostate cancer (CRPC) and the formation of metastatic disease represent major clinical unmet needs in the treatment of PCa. The current treatment for CRPC includes the use of the cytotoxic agent docetaxel. Docetaxel belongs to the taxane class and binds to the microtubules. Hereby, docetaxel stabilizes the microtubules and prevents tubulin depolymerization, resulting inhibition of cell proliferation. Unfortunately, clinical responses to docetaxel are modest since a subset of patients does not respond to docetaxel, develops adverse effects or acquires resistance to the docetaxel treatment (2). Therefore, novel treatment strategies for (therapy-resistant) PCa are urgently needed.

Epidemiological studies have revealed a reduced incidence of different types of cancer, including PCa, in schizophrenic patients (3–5). This suggests that the use of antipsychotics could protect against the development of cancer. These findings were subsequently further reinforced by several meta-analyses (6, 7). Penfluridol is a long-acting oral antipsychotic drug and is prescribed to treat chronic schizophrenia and other psychiatric disorders (8–11). Interestingly, multiple preclinical studies have demonstrated that penfluridol exerts cytotoxic effects in bladder, breast, colon and pancreatic cancer preclinical models (12–16). To date, the effect of penfluridol on human PCa cells remains elusive. In this study, we have investigated the anti-tumor effects of penfluridol in preclinical human PCa models, including monolayers and three-dimensional cell cultures and *ex vivo* cultured PCa tissue slices. Finally, we have tested the effects of penfluridol in docetaxel-resistant PCa cells *in vitro* and have examined the effect of penfluridol in combination with docetaxel in these docetaxel-resistant PCa cells.

## 2 Material and methods

### 2.1 Two- and three-dimensional cultures

Human PCa cells lines PC3, PC-3M-Pro4luc2, DU145, 22Rv1 and C4-2B4 were cultured in monolayers as described in **Supplementary Table 1**. Docetaxel-resistant PCa cell lines PC3-DR, DU145-DR and 22Rv1-DR were generated by treatment of the cells with increasing concentrations of docetaxel (17, 18). Three-dimensional cultures were generated from PC3 cells and MSK-PCa1 cells, both derived from PCa bone metastasis, and the PCa liver metastasis model NM60 (19–21).

### 2.2 Viability assays

1,500 human PCa cells were seeded per well in 150 μl medium in 96-well plates. After 24 hours, the cells were treated with a dose-range of penfluridol (Sigma-Aldrich, Saint Louis, MO, USA P3371, RRID: SCR_008988) or vehicle (ethanol in medium). The medium was refreshed as indicated and the viability was measured after 72 hours. To investigate the effect of penfluridol in combination with docetaxel on docetaxel-resistant PCa, docetaxel-resistant PCa cells were exposed to a dose-range of docetaxel (Sigma-Aldrich) in combination with one concentration of penfluridol for 72 hours. After 72 hours, 20 μl of 3-(4,5 dimethylthiazol-2-yl)-5-(3-carboxymethoxyphenyl)-2-(4-sulfophenyl)-2H-tetrazolium (MTT) (Promega, Madison, WI, G3581, RRID: SCR_006724) was added to the culture medium and mitochondrial activity was measured after 2 hours (SpectraMax iD3, Molecular Devices).

Three-dimensional cultures of PC3, MSK-PCa1 and NM60 cells were treated with a dose-range of penfluridol. After 3 days, the viability of the cultures was determined using the Cell Titer Glo assay (Promega, Madison, WI, G9681). In parallel to the viability assays, histology was performed on three-dimensional cultures by executing H&E and immunofluorescent stainings for cleaved caspase-3, pancytokeratin and PCNA (**Supplementary Table 2**) (21).

### 2.3 Caspase-3/7 assay

1,500 human PCa cells were seeded in 150 μl medium. After overnight incubation, the cells were exposed to penfluridol for 2 hours. Human PCa cells exposed to 1 μM staurosporine for 24 hours were used as a positive control. The caspase-3/7 activity was measured by performing the Caspase-Glo^®^ 3/7 Assay System according to the manufacturer’s protocol (Promega). Luciferase activity was measured after 30 minutes with a luminometer (Spectramax iD3, Molecular Devices).

### 2.4 Clonogenic assay

Hundred human PCa cells were seeded in 2 ml of medium in a 6-well plate. After 24 hours, cells were stimulated with penfluridol for 2 hours. Colonies were fixed with 4% paraformaldehyde and stained with a 0.2% crystal violet solution after 15–20 days. The number of colonies was counted and the Colony Area Plugin for ImageJ was used to quantify the average colony area.

### 2.5 Aldefluor assay

PCa cells were treated with a dose-range of penfluridol for 2 hours. After 48 hours, 10^6^ cells were collected for the Aldefluor Assay. The Aldefluor assay was performed by using the ALDEFLUOR Assay Kit (StemCell Technologies, Vancouver, Canada, #01700, RRID;SCR_013642) (22). The ALDH substrate was added to the collected cells, resulting into intracellular conversion of the substrate by intracellular ALDH into a fluorescent product. The percentage of ALDH^high^ stem/progenitor-like cells was determined by FACS analysis (LSRII, BD Biosciences, Franklin Lakes, NJ, USA) (24). The percentage ALDH^high^ cells was analyzed by using FlowJo10.0 by measuring the percentage after doublet exclusion and compared to DEAB controls.

### 2.6 *Ex vivo* tissue slice culture and scoring

PCa tumor tissue was obtained from cell line-derived xenografts (CDX) and previously established patient-derived xenograft (PDX) models (21). In addition, primary prostate tumor material was obtained by transurethral resection of the prostate (TURP) after informed consent (Pronet p05.85 and RBUT-ID-PROSTAAT-151). Additional (clinical) details are shown in **Supplementary Table 3**.

PCa tumor tissue were sliced and cultured as previously described (23). After one day, the PCa tissue slices were treated with 100 μM penfluridol. After exposure to penfluridol for 3 days, the PCa tissue slices were fixed, embedded in paraffin and sectioned (15, 23). Paraffin sections were stained with H&E and immunofluorescent stainings for cleaved caspase-3, pancytokeratin and PCNA were performed in parallel (see **Supplementary Table 2**). Images were captured using the SP8 confocal microscope (Leica) and the Midi Panoramic slide scanner (3D Histech) (15). The effect of penfluridol on the PCa tissue slices was quantified as previously described (15). The necrotic area and positive cleaved caspase-3 area were quantified by using ImageJ software (National Institutes of Health). Furthermore, sections were scored based on tissue integrity (H&E staining), the presence of fragmented cytokeratin, proliferating cells (PCNA), and apoptosis (cleaved caspase-3). The average cumulative scores of four sections are displayed in heatmaps, where a higher score indicates a decrease in tumor tissue quality (14).

### 2.7 Statistical analyses

Statistical analyses were performed by using GraphPad Prism, version 10.2.3. One-way ANOVA with a Bonferroni *post-hoc* test was performed to test for statistical differences in the *in vitro* viability experiments. An unpaired t-test was performed to test for statistical differences in caspase-3/7 apoptosis assays. IC50-values were calculated by using non-linear regression in combination with the dose-response-inhibition equation with four parameters in the GraphPad Prism software package. Two-way ANOVA with a Bonferroni *post-hoc* test was used to test for statistical difference in the docetaxel-resistance cell lines. The Bliss independence model (C_e_ = A + B – A x B) was used to calculate the expected effect (C_e_) of penfluridol in combination with docetaxel. The combination index (CI) was calculated by dividing the observed effect (C_o_) to the expected effect (C_e_). A CI larger than 1 indicates synergy.

* p<0.05, ** p<0.01, *** p<0.001 and **** p<0.0001

## 3 Results

### 3.1 Penfluridol treatment decreases the viability, induces apoptosis and reduces cancer stem cell phenotype in human PCa cells *in vitro*

**Figure 1:**
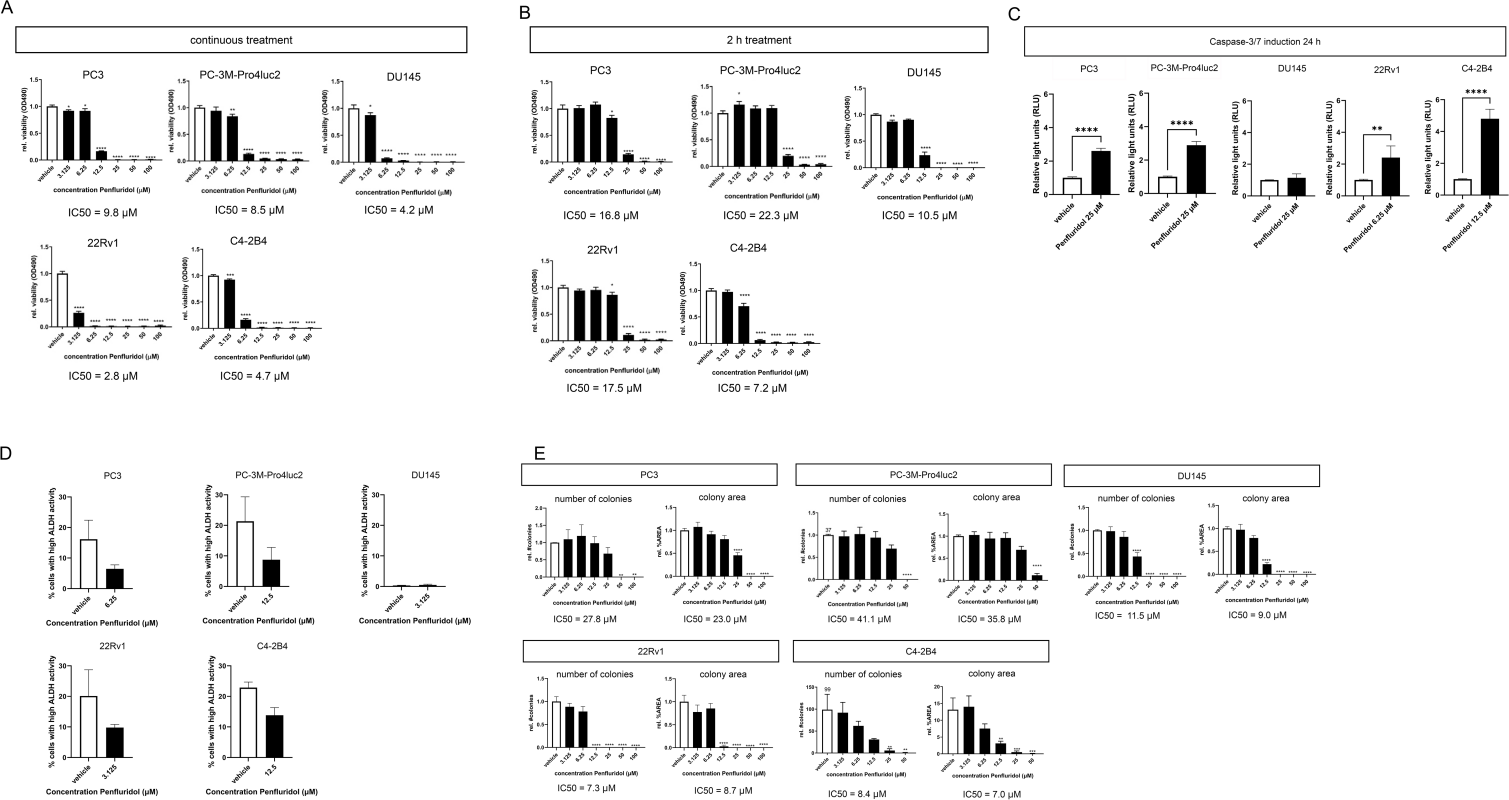
Penfluridol reduces viability, induces apoptosis and reduces stemness of human PCa cells *in vitro.* Continuous **(A)** and two-hour **(B)** exposure of the human PCa cell lines PC-3M-Pro4luc2, DU145, 22Rv1 and C4-2B4 to a dose-range of penfluridol resulted in a reduced viability after 72 hours. **(C)** Treatment with penfluridol resulted in an increase of caspase-3/7 levels after 24 hours in PC-3M-Pro4luc2, 22Rv1 and C4-2B4 cells. **(D)** Exposure to penfluridol reduced the percentage of cells with high ALDH activity (ALDH^high^) after 48 hours in multiple PCa cell lines. **(E)** Treatment of human PCa cell lines with penfluridol significantly decreased the number of colonies and colony area. Mean +/- standard error of the mean (SEM) (n=3) * p<0.05, ** p<0.01, *** p<0.001 **** p<0.0001, one-way ANOVA (viability, clonogenic assay) and two-sided t-test (caspase-3/7 induction).

To investigate the effects of penfluridol on the viability of human PCa cells, PC3, PC-3M-Pro4luc2, DU145, 22Rv1 and C4-2B4 cells were exposed to a dose-range penfluridol for 72 hours. Viability assays indicated a significantly reduced viability after treatment with 3.125 μM penfluridol in PC3, 6.25 μM penfluridol in PC-3M-Pro4luc2 (p<0.01) and 3.125 μM penfluridol in DU145 (p<0.05), 22Rv1 (p<0.0001) and C4-2B4 (p<0.001) cells (**Figure 1A**). The IC50 values ranged from 2.8-9.8 μM penfluridol treatment. A short penfluridol exposure of 2 hours significantly reduced the viability of PC3 cells, (p<0.05 12.5 μM, IC50 = 16.8 μM), PC-3M-Pro4luc2 cells (p<0.0001 25 μM, IC50 = 22.3 μM), DU145 cells (p<0.0001 12.5 μM, IC50 = 10.5 μM), 22Rv1 cells (p<0.05 12.5 μM, IC50 = 17.5 μM) and C4-2B4 cells (p<0.0001 6.25 μM, IC50 = 7.2 μM) cells after 72 hours (**Figure 1B**, **Supplementary Figure 1A**). The effect of penfluridol on caspase-3/7 induction was investigated in a panel of human PCa cells. Penfluridol significantly increased caspase3/7 levels in PC3 cells (p<0.0001), PC-3M-Pro4luc2 cells (p<0.0001), 22Rv1 (p<0.01) and C4-2B4 cells (p<0.0001) after 24 hours (**Figure 1C** and **Supplementary Figure 1B**). To examine the effect of penfluridol on the PCa stem/progenitor subpopulation, changes in the percentage ALDH^high^ cells were measured by performing an Aldefluor assay. Previous research by our group has shown that PCa cells with high ALDH activity are associated with elevated clonogenicity and invasiveness *in vitro* and increased tumor progression and metastasis formation *in vivo* (22, 24).The percentage of ALDH^high^ subpopulation of PCa stem/progenitor cells was reduced upon treatment with penfluridol after 48 hours (**Figure 1D**) In line with these findings, clonogenic assays revealed a dose-dependent reduction in number of colonies and colony area in human PCa cells exposed to penfluridol (**Figure 1E**, **Supplementary Figure 1C**).

### 3.2 Penfluridol treatment decreases the viability, induces apoptosis in ‘near-patient’ human PCa models

**Figure 2:**
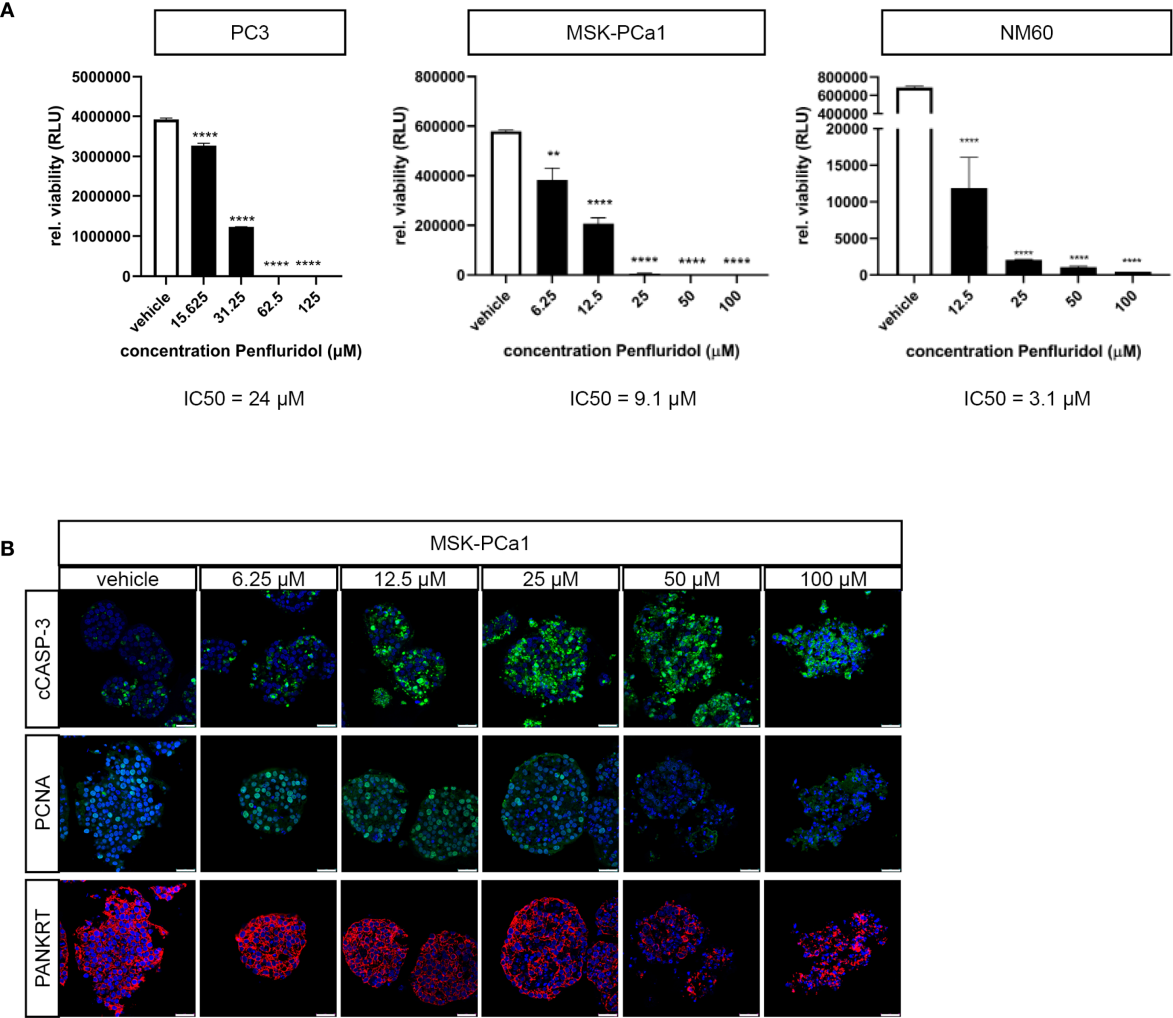
Penfluridol displays anti-tumor effects in three-dimensional cell cultures of advanced human PCa. Three-dimensional cell cultures were generated from PC3 cells, PCa bone metastases (MSK-PCa1) and PCa liver metastases (NM60) and exposed to a dose-range of penfluridol for 72 hours. **(A)** Viability assays revealed a significant dose-dependent decrease in viability after treatment with penfluridol. Mean +/- standard error of the mean (SEM), ** p<0.01, **** p<0.0001, one-way ANOVA (n=3). **(B)** Representative confocal images of three-dimensional MSK-PCa1 cell cultures stained for apoptosis (cleaved caspase-3, cCASP-3 in green), proliferation (proliferating cell nuclear antigen, PCNA in green), epithelial cell marker (pancytokeratin PANKRT in red) and combined with nuclear staining (DAPI, in blue) indicated decreased cancer cell proliferation and integrity upon penfluridol exposure. Magnification 63x, scale bar = 25 μm.

Next, the effect of penfluridol was examined in advanced ‘near-patient’ PCa models, including three-dimensional cultures and *ex vivo* cultured tumor tissue slices (23, 25). Three-dimensional cultures of PC3 cells, MSK-PCa1 cells [PCa bone metastases material (22)] and NM60 cells [PCa liver metastasis PDX model (20, 21)] were exposed to a dose-range of penfluridol for 72 hours. Treatment with penfluridol significantly and dose-dependently reduced the viability of PCa tumoroids (**Figure 2A**). Immunohistochemical analyses of MSK-PCa1 confirmed a reduction in the proliferation marker PCNA and fragmentation of epithelial protein pancytokeratin (panKRT) upon penfluridol exposure. Furthermore, apoptosis was induced (cleaved caspase-3, cCASP-3) and a complete loss of organoid architecture was observed in MSK-PCa1tumoroids (**Figure 2B**). Overall, these results suggest that penfluridol has anti-tumor effects in three-dimensional cultures of human PCa.

**Figure 3:**
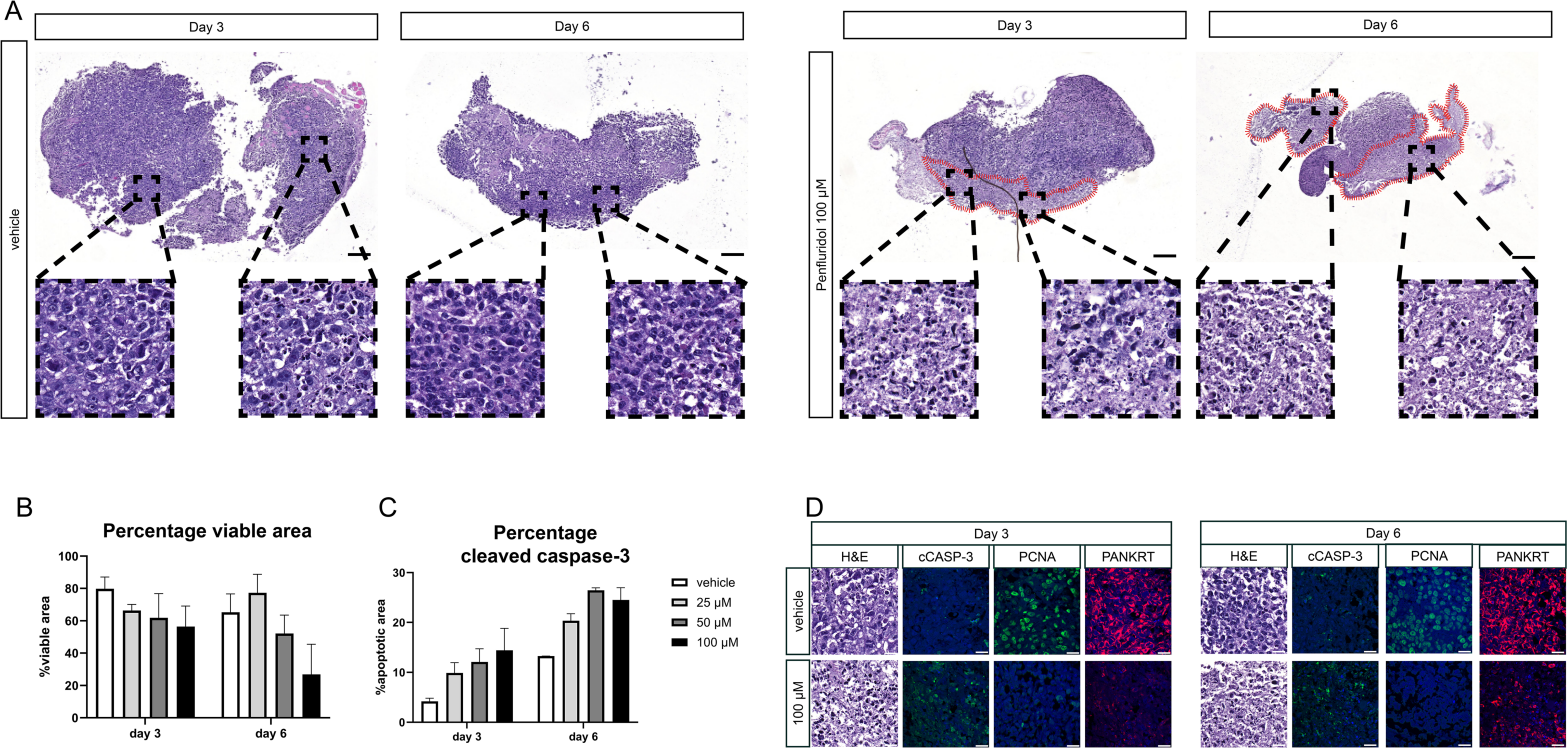
Penfluridol induces cancer cell death in cell line-derived PCa tissue slices. PCa tissue slices were generated from a tumor derived from a human PCa cell line derived xenograft (CDX) (PC-3M-Pro4luc2). Tumor tissue slices were subsequently treated with penfluridol for 3 and 6 days. **(A)** H&E staining revealed a lower cell density and the presence of fragmented nuclei in the outer rim of penfluridol-treated tissue slices (red marked areas) after 3 and 6 days. Magnification 4x, scale bar = 200 μm. **(B)** The total viable area in penfluridol treated tissue slices was quantified using ImageJ. This indicated a decrease in the total viable area upon penfluridol treatment. **(C)** Quantification of the total positive cleaved caspase-3 area by ImageJ showed a dose-dependent elevation. **(D)** Representative images of *ex vivo* cultures tumor tissue slices stained for H&E, apoptosis (cleaved caspase-3, cCASP-3 in green), proliferation (proliferating cell nuclear antigen, PCNA in green), epithelial cell integrity (pancytokeratin PANKRT in red) and nuclei (DAPI, in blue) indicated an anti-tumor response after exposure to 100 μM penfluridol. Magnification 63x, scale bar = 25 μm Tumor tissue slices treated with penfluridol were scored based on tissue quality (H&E staining), loss of proliferation (PCNA), induction of apoptosis (cleaved caspase-3) and the presence of fragmented cytokeratin (15). **(D)** Cumulative scores of four sections were calculated and displayed in heatmaps where a higher score indicates a decrease in tissue quality. Scoring of PC-3M-Pro4luc2 tissue slices revealed an increase in the cumulative score upon treatment with penfluridol, indicating overall reduced tissue quality.

**Figure 4:**
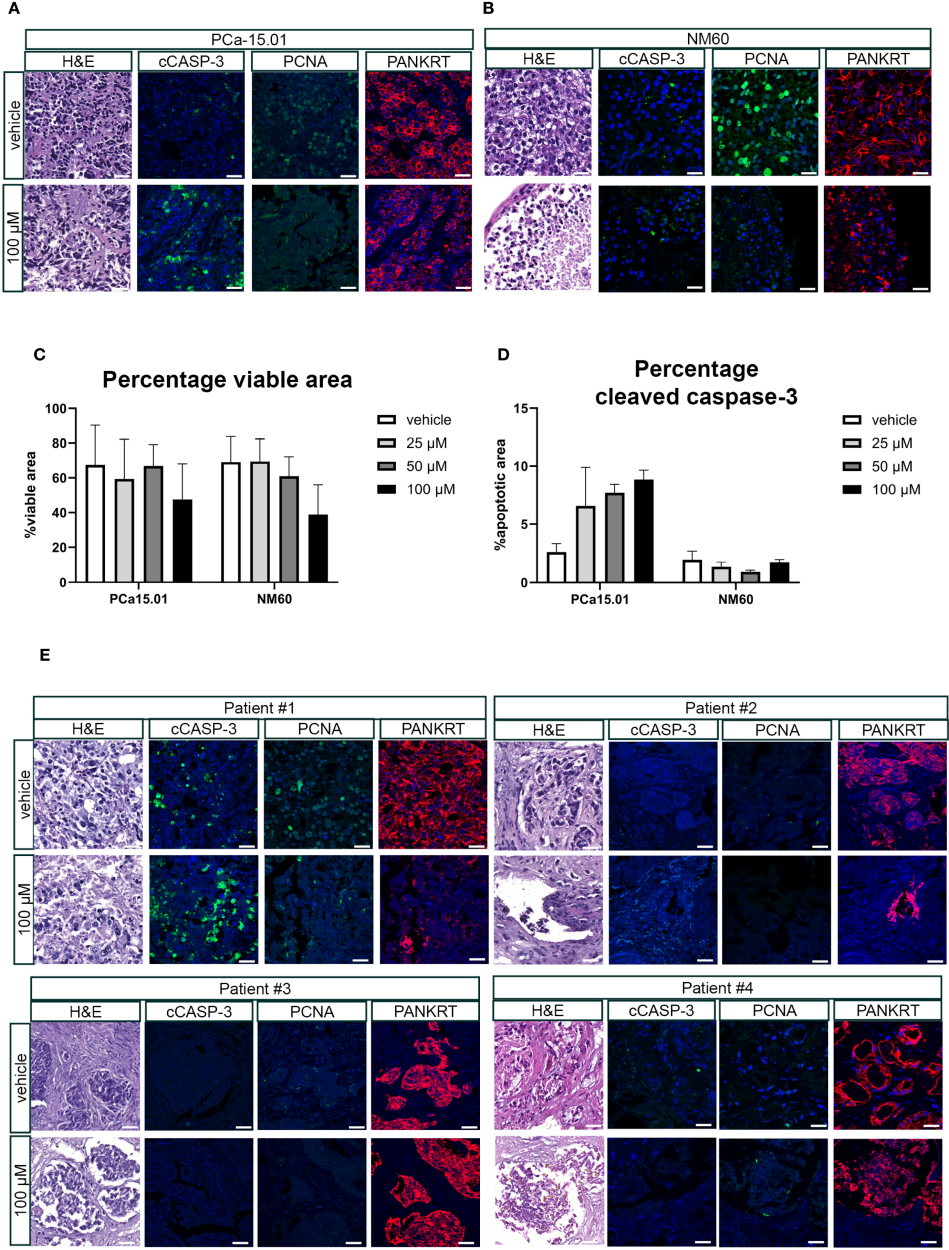
Penfluridol displays anti-tumor effects in *ex vivo* cultured tumor tissue slices from PCa patient-derived xenograft models and primary biopsy samples. Tissue slices were generated from patient-derived xenograft (PDX) models PCa-15.01 and NM60 (21) **(A–D)** or primary patient biopsies **(E)**. *Ex vivo* treatment of tissue slices resulted in the induction of an anti-tumor response as indicated by a reduced total viable area in PCa-15.01 and NM60 tissue slices **(C)** and an induction of cleaved caspase-3 levels in PCa15.01 tissue slices **(D)** after 3 days of treatment. Green = PCNA, Red = PANKRT, blue = DAPI. Magnification 63x, scale bar = 25 μm.

PCa tissue slices were generated from a subcutaneously growing cell-derived xenograft (CDX) of PC-3M-Pro4luc2 cells and cultured in the presence of 100 μM penfluridol for 3 or 6 days. H&E staining revealed a lower tumor cell density and the presence of fragmented nuclei in the outer rim of penfluridol-treated tissue slices (red marked areas in **Figure 3A**). Quantification of the percentage total viable area in H&E-stained sections revealed a decrease in viability upon penfluridol treatment (**Figure 3B**). In line with these findings, quantification of the positive cleaved caspase-3 area indicated a dose-dependent increase in cleaved caspase-3 levels upon penfluridol treatment (**Figure 3C**). Histological evaluation by using immunofluorescence and confocal microscopy indicated a reduction in the number of proliferating cells and an increase in tumor cell apoptosis and fragmented cytokeratin upon treatment with penfluridol (**Figure 3D**). The effect of penfluridol was quantified by using a scoring system based on the loss of tissue architecture, the absence of proliferating cells and the presence apoptotic cells and fragmented cytokeratin (15). Scoring of the tissue slices revealed a slight increase in the score after penfluridol treatment (**Supplementary Figure 2A**). Tissue slices were generated from our previously established patient-derived xenograft (PDX) models PCa-15.01 and NM60. These PDX models were derived from a hormone-naïve PCa patient (PCa-15.01) or from a patient with mCRPC (NM60). Tumor tissue slices were treated with 100 μM penfluridol for three days (**Figures 4A, B**) (21, 23). Treatment with penfluridol resulted in a decreased total viable area (**Figure 4C**), elevated levels of cleaved caspase-3 (**Figure 4D**), decreased numbers of proliferating tumor cells and loss of tumor cell integrity leading to an overall increase in tissue score (**Supplementary Figure 2B**). These observations indicate that penfluridol displays anti-tumor properties in these *ex vivo* cultured tumor tissue slices derived from PCa PDX models. Finally, similar anti-tumor effects of penfluridol were found in tissue slices derived from freshly isolated PCa biospies using the same experimental setup (**Figure 4E**). Taken together, these results suggest that penfluridol treatment can induce an anti-tumor response in *ex vivo* cultured PCa tissue slices.

### 3.3 Penfluridol induces cell death in chemotherapy-resistant PCa cells *in vitro* and sensitizes docetaxel-resistant PCa cells to docetaxel

**Figure 5:**
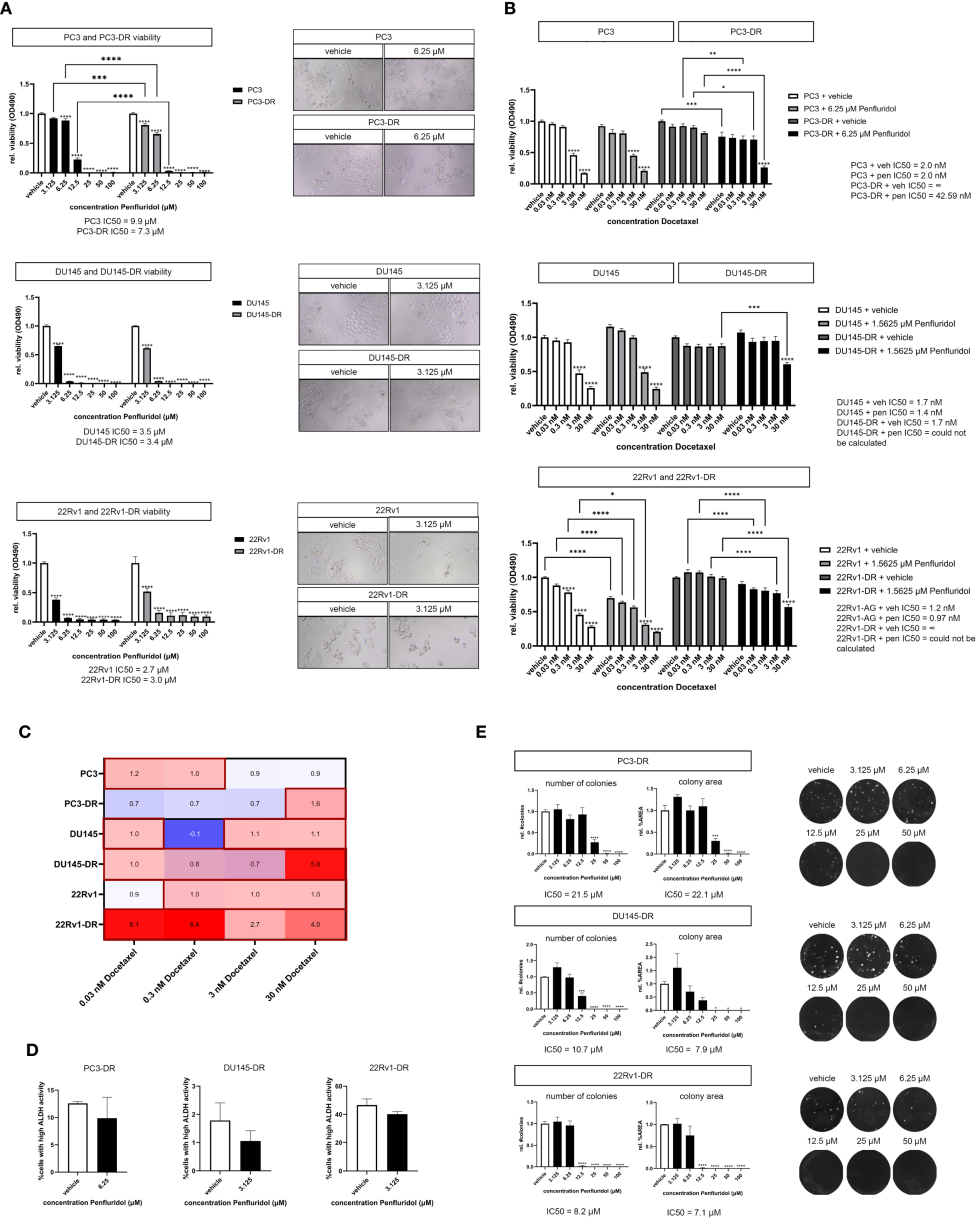
Penfluridol reduces the viability of docetaxel-resistant PCa cells and induces synergistic effects in combination docetaxel in docetaxel-resistant PCa cells. **(A)** Docetaxel-resistant (-DR) PCa cells PC3-DR, DU145-DR, 22Rv1-DR were exposed to a dose-range of penfluridol for 72 hours. Penfluridol significantly reduces the viability in both docetaxel-resistant cell lines PC3-DR, DU145-DR, 22Rv1-DR and docetaxel-sensitive PC3, DU145 and 22Rv1 cells. The reduction in viability in PC3-DR cells was more pronounced when compared to the parental PC3 cell line after treatment with 3.125, 6.25 and 12.5 μM penfluridol. Mean +/- standard error of the mean (SEM), ** p<0.01, **** p<0.0001, two-way ANOVA (n=3). **(B)** Human PCa cells were exposed to dose-range of docetaxel in combination with a low dose penfluridol for 72 hours. Administration of a low dose of penfluridol (i.e. 6.25 μM in PC3(-DR) and 1.5625 μM in DU145(-DR) and 22Rv1(-DR)) significantly reduced the viability of docetaxel-resistant cell lines, indicating synergistic effects of penfluridol in combination with docetaxel in these cell lines. **(C)** By using the Bliss independence model (C= A+B-AxB), the predicted effect of the combination therapy C was calculated where A represents the effect of penfluridol monotherapy and B is the effect of docetaxel monotherapy. Subsequently, CI was calculated by dividing the observed effect of the combination therapy by the predicted effect of the combination therapy, where a CI higher than 1 indicates synergy. The combination of penfluridol and docetaxel treatment in docetaxel-resistant cell lines PC3-DR, DU145-DR and 22Rv1-R resulted in a CI larger than 1, indicating synergistic effects of penfluridol in combination with docetaxel. **(D)** Exposure to penfluridol slightly reduced the percentage of cells with high ALDH activity (ALDH^high^) cells after 48 hours in multiple docetaxel-resistant PCa cells. **(E)** Treatment of human docetaxel-resistant PCa cell lines with penfluridol significantly decreased the number of colonies and colony area. Mean +/- standard error of the mean (SEM) (n=3) * p<0.05, ** p<0.01, *** p<0.001 **** p<0.0001, one-way ANOVA (clonogenic assay), two-way ANOVA (viability assay).

The development of therapy resistance, including resistance to the chemotherapeutic agent docetaxel, represents an important clinical unmet need in the treatment of PCa patients. Docetaxel-resistant PCa cell lines PC3-DR, DU145-DR and 22Rv1-DR were exposed to penfluridol *in vitro.* Penfluridol significantly reduced the viability of PC3-DR, DU145-DR and 22Rv1-DR cells after 72 hours (**Figure 5A**). Strikingly, penfluridol induced a more pronounced anti-tumor effect in PC3-DR cells than in docetaxel-sensitive PC3 cells (p<0.001 at 3.125 μM and p<0.0001 at 6.25 μM). This was also reflected by a lower IC50 value of PC3-DR cells compared to PC3 i.e. 7.3 μM in PC3-DR cells compared to 9.9 μM in PC3 cells.

Next, we investigated the effects of penfluridol in combination with docetaxel on docetaxel-resistant PCa cells. The viability of docetaxel-resistant PC3-DR, DU145-DR and 22Rv1-DR cells was significantly decreased when docetaxel was administered in combination with a low dose of penfluridol (**Figure 5B**, **Supplementary Figures 3A–D**). The Bliss independence model (C= A + B – A x B) was used to calculate the combination index (CI). The CI was calculated by dividing the predicted inhibition C by the observed inhibition, where a CI > 1 indicates synergy (26). The combination of penfluridol and docetaxel treatment in the docetaxel-resistant cell lines PC3-DR, DU145-DR and 22Rv1-DR resulted in a stronger reduction in viability when similar dosages of docetaxel and penfluridol were administered separately (**Supplementary Figures 2B–D**) and induced synergistic effects in these cell lines, as indicated by the combination index (**Figure 5C**). Moreover, penfluridol reduced the percentage ALDH^high^ cells of docetaxel-resistant PCa cells after 48 hours (**Figure 5D**) and significantly decreased the number of colonies and colony area of docetaxel-resistant PCa cells (**Figure 5E**). These results suggest that administering a low dose of penfluridol induces anti-tumor effects and might sensitize docetaxel-resistant PCa cells to docetaxel.

## 4 Discussion

Penfluridol was discovered in 1968 and is an oral antipsychotic drug with a long half-life (8–10). Recently, cationic amphiphilic drugs (CADs), including penfluridol, have drawn substantial attention for their anti-neoplastic properties in different tumor types. However, the effects of CADs, including penfluridol, on PCa remain unclear. In this study we found that penfluridol induces anti-tumor effects in multiple preclinical PCa models including ‘near-patient’ patient-derived tumor models such as three-dimensional cultures and *ex vivo* cultured tumor tissue slices (14, 23–25). Moreover, our study reports for the first time that penfluridol displays anti-tumor effects in chemotherapy-resistant PCa and that penfluridol induces synergistic effects in combination with docetaxel in docetaxel-resistant PCa cells.

Docetaxel is the first-line therapy for patients suffering from metastatic CRPC (mCRPC). However, more than 50% of all patients do not respond to docetaxel and patients who respond eventually develop resistance to docetaxel (27). Unfortunately, the exact molecular mechanisms responsible for docetaxel-resistance are currently unknown. Studies have suggested that induction of epithelial-to-mesenchymal transition (EMT) and increased stemness are associated with docetaxel-resistance in PCa (18). Increased stemness and cancer-stem cells are associated with a poor prognosis in human PCa (28). A previous study revealed that penfluridol reduced renal cell carcinoma growth by inhibiting stemness (29). In line with these findings, we observed that penfluridol reduced the percentage of ALDH^high^ PCa cells, thereby suggesting that penfluridol can reduce PCa stem cells *in vitro*. Moreover, penfluridol decreased the viability of docetaxel-resistant PCa cells *in vitro.* These findings are in accordance with those of a previous study reporting that penfluridol can target paclitaxel-resistant breast cancer cells and that penfluridol can inhibit microtubule polymerization (30, 31). Future studies that examine the working mechanism of penfluridol in docetaxel-resistant prostate cancer cells are needed. Furthermore, our study reports for the first time that penfluridol exerts synergistic effects with docetaxel in chemotherapy-resistant PCa cells. The results of this study indicated that penfluridol may be a novel therapeutic option for docetaxel-resistant PCa cancer.

Since penfluridol is a clinically-approved agent, the pharmacokinetics, safety and toxicity are well-known (10). Therefore, repurposing of penfluridol for the treatment of PCa might represent a time- and cost-effective approach (32). Future preclinical *in vitro* and *in vivo* studies both on the anti-tumor effects and mechanism of action of penfluridol, may facilitate the clinical translation of penfluridol or related compounds. Clinical studies are required to elucidate which subgroup of patients with PCa will benefit the most from penfluridol treatment. Such studies should may also encompass the putative adverse effects of the neuropsychiatric drug penfluridol in prostate cancer patients. Our findings suggest that penfluridol is a potent anti-tumor agent in advanced PCa, including mCRPC and docetaxel-resistant prostate cancer. Clinical phase II studies investigating the effects of systemic treatment with penfluridol in advanced PCa patients are needed. The described *ex vivo* culture models could help in further deciphering which subgroup of patients will benefit the most from penfluridol treatment, although further co-clinical studies are necessary to evaluate the predictive value of these cultures. Taken together, we have identified penfluridol as a promising anti-cancer agent by causing cytolytic effects in multiple preclinical models of human PCa. We believe that repurposing of penfluridol might represent an interesting option for the treatment of advanced PCa.

## Data Availability

The raw data supporting the conclusions of this article will be made available by the authors, without undue reservation.
