# Supplementary figures and images for: Anti-neoplastic effects of the antipsychotic drug penfluridol in preclinical prostate cancer models
Source: Front Oncol. 2025 Oct 14;15:1685758. doi: 10.3389/fonc.2025.1685758 (PMC12558770; doi:10.3389/fonc.2025.1685758)

A

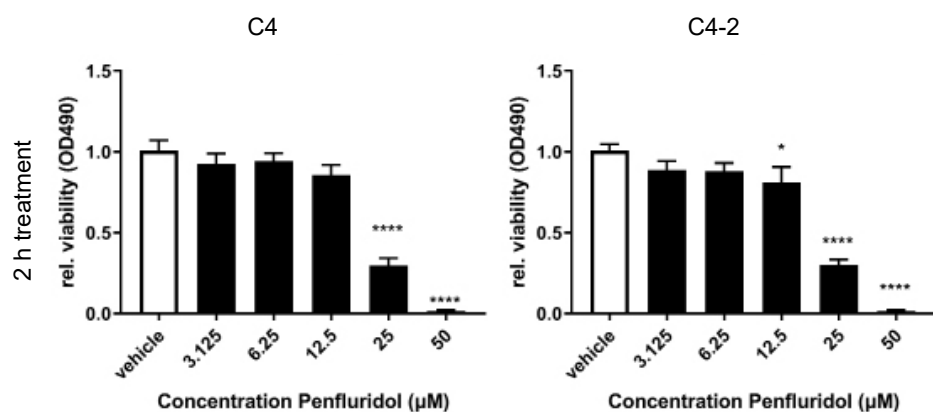

B

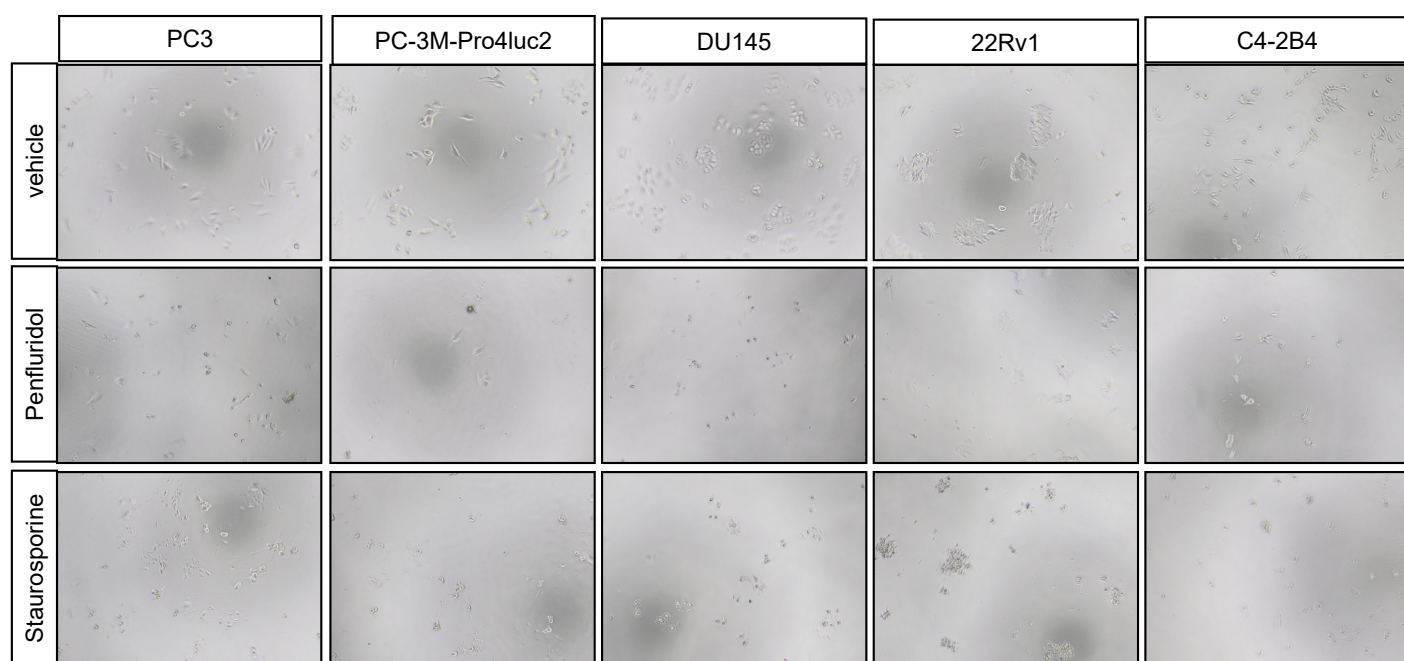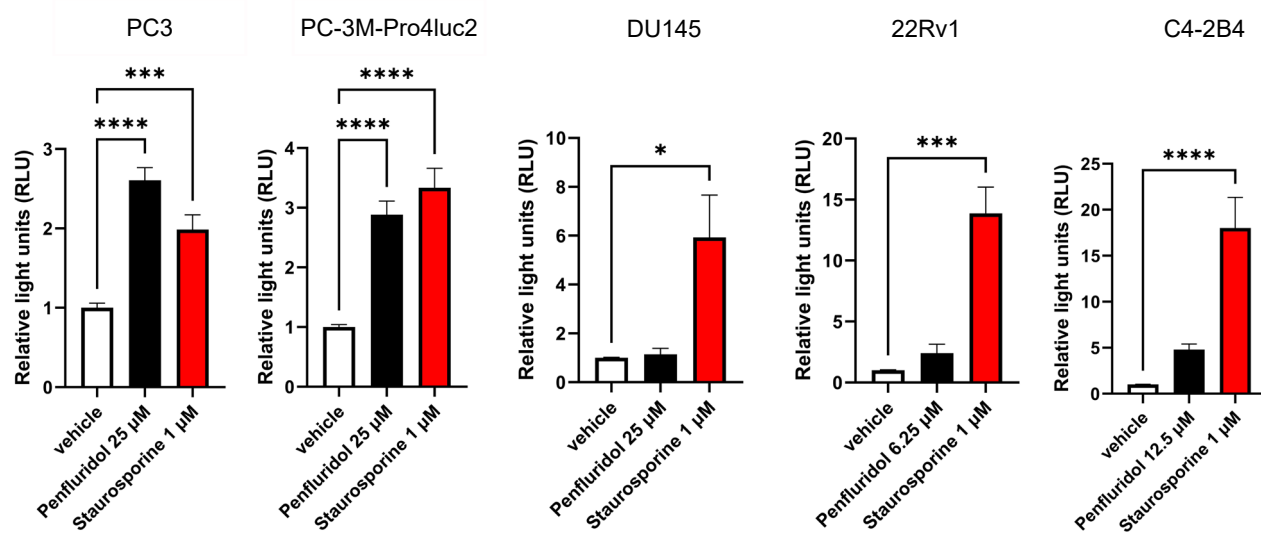

S. Figure 1

C

PC3

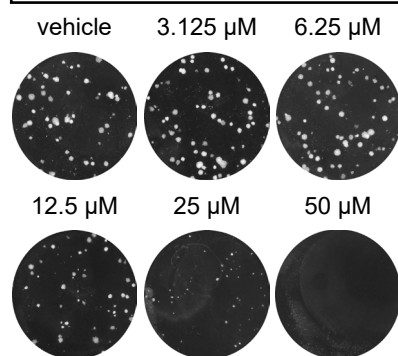

22Rv1

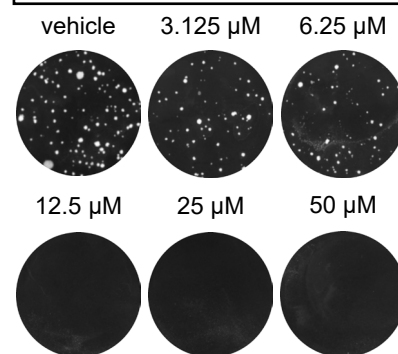

PC-3M-Pro4luc2

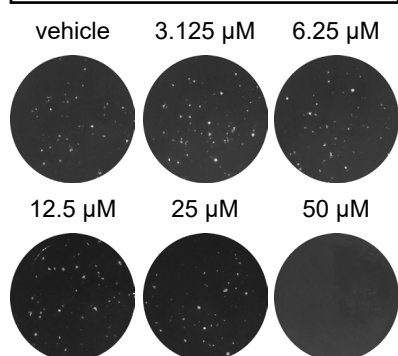

C4-2B4

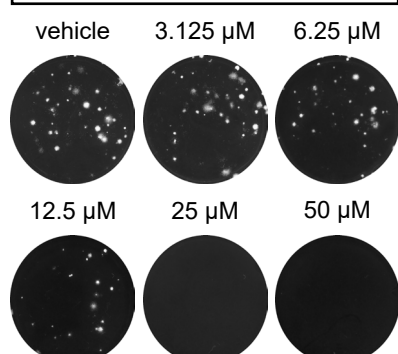

DU145

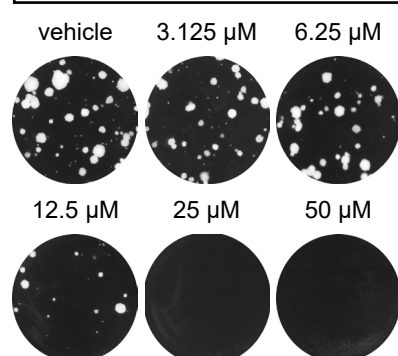

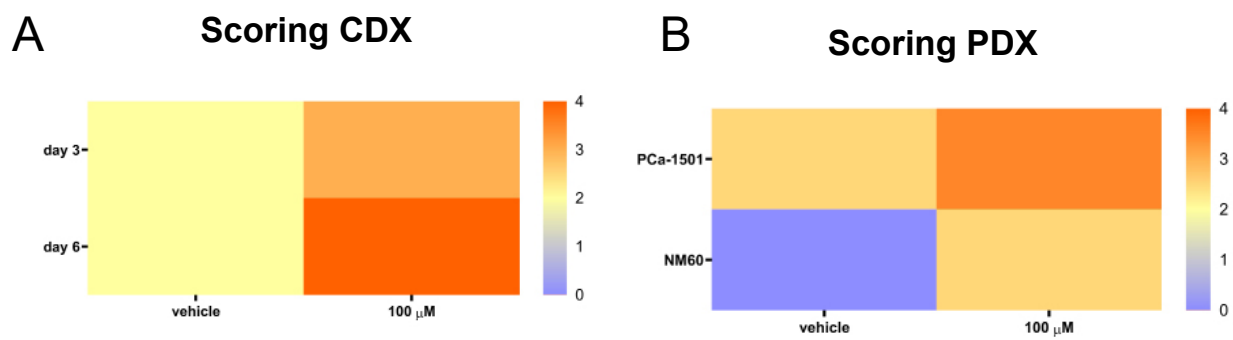

S. Figure 2

A

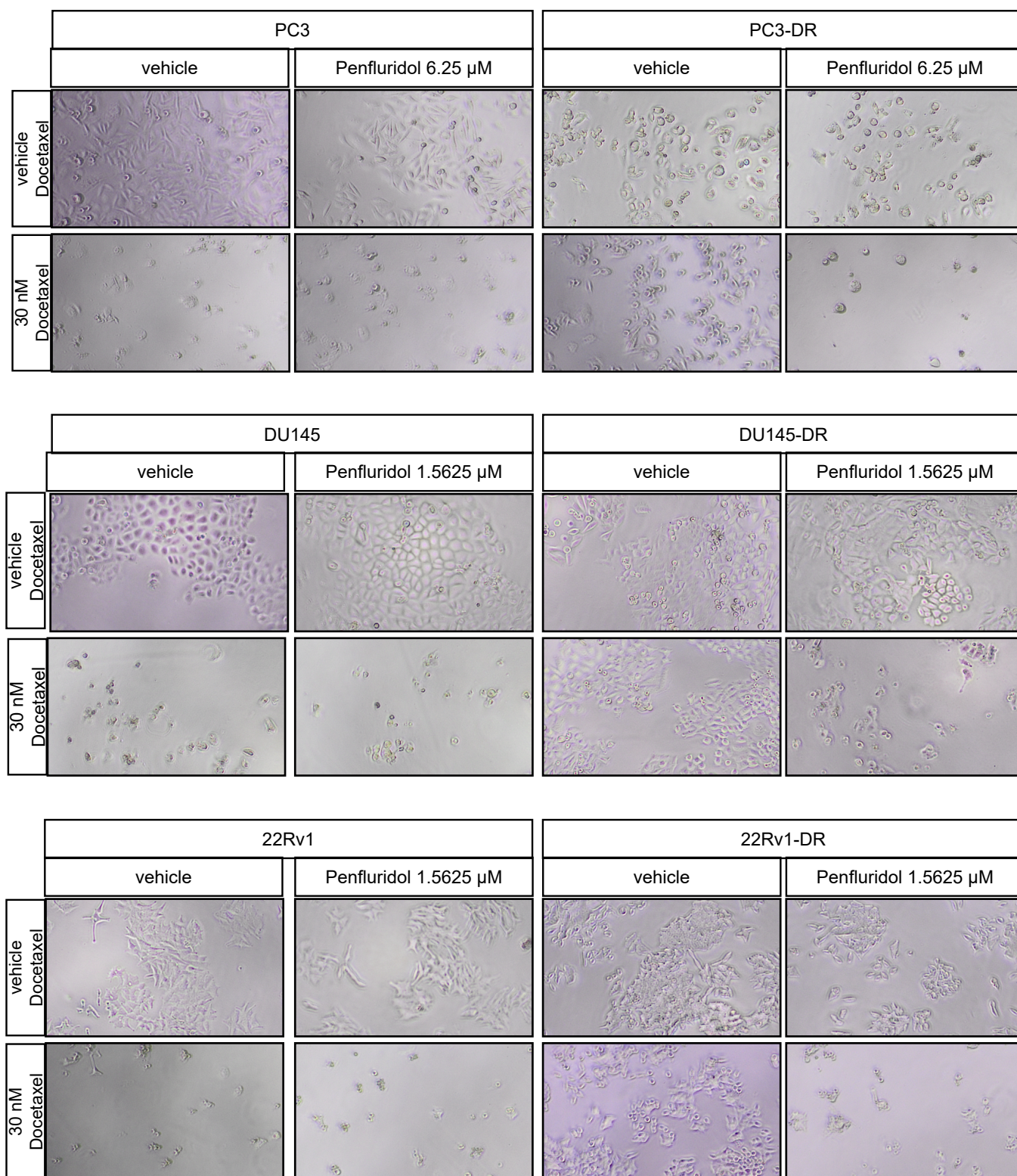

S. Figure 3

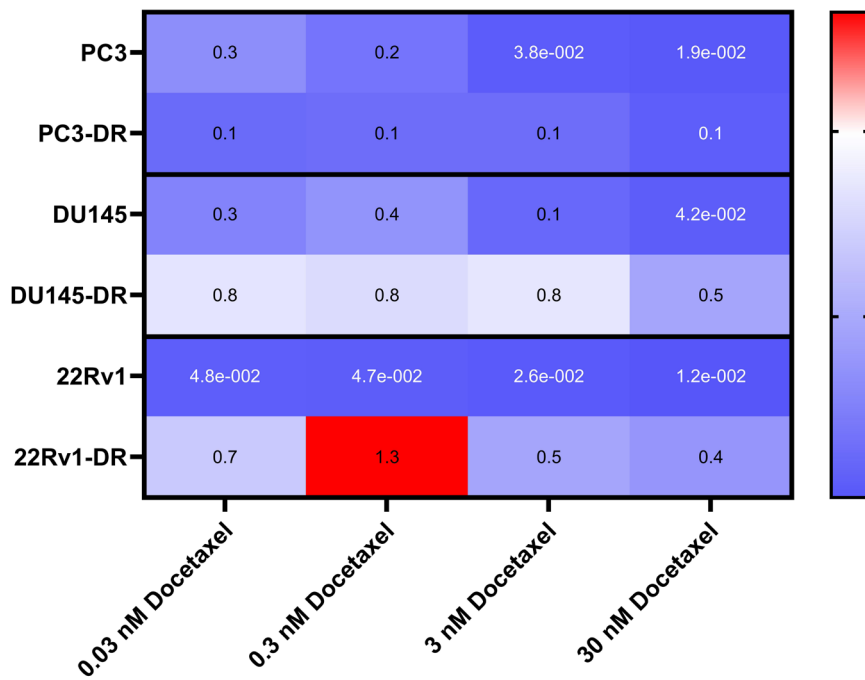

B

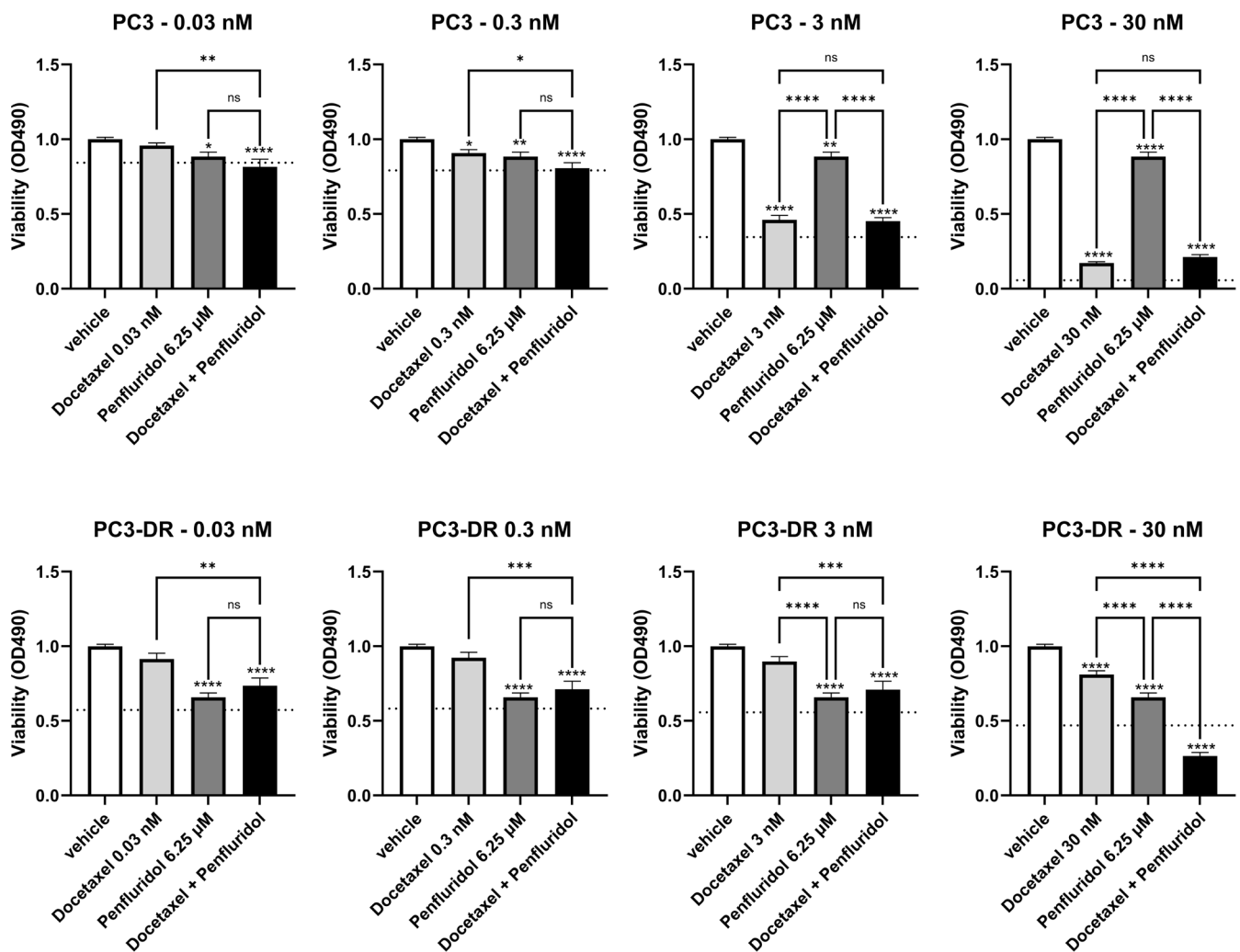

S. Figure 3

C

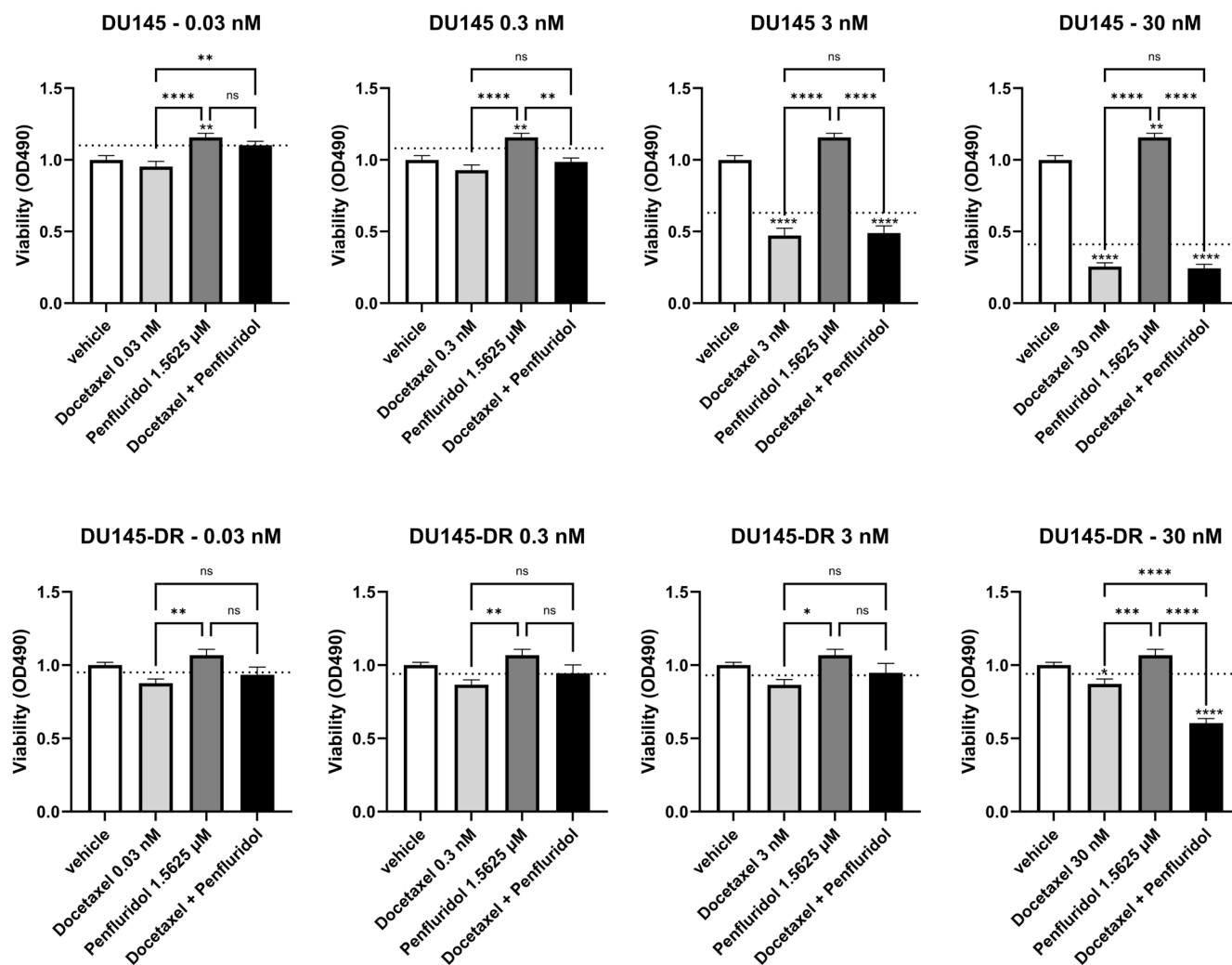

S. Figure 3

D

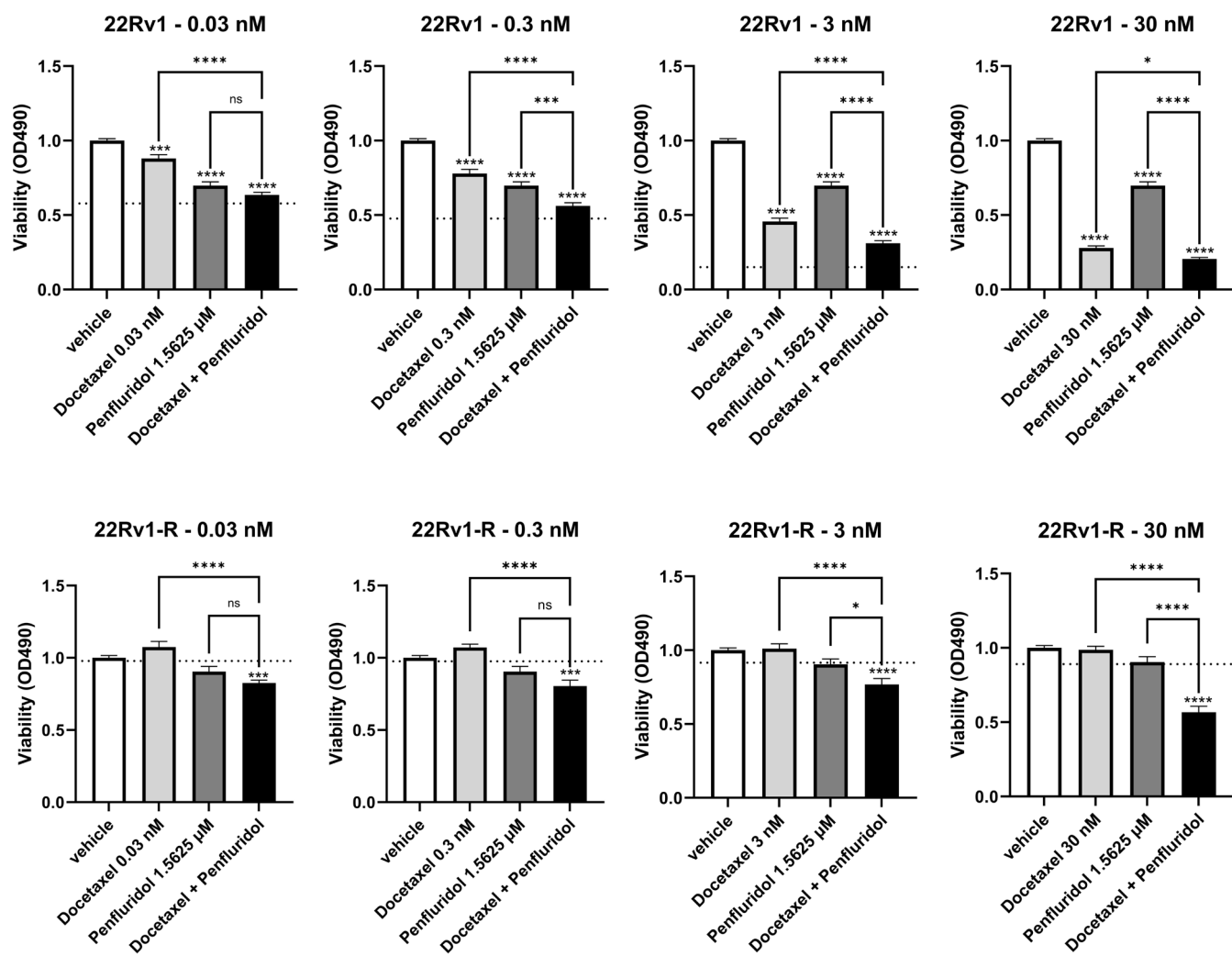

S. Figure 3

Supplement: Supplementary file 1 [file DataSheet1.pdf]
