# Supplementary material for: Anti-neoplastic effects of the antipsychotic drug penfluridol in preclinical prostate cancer models
Source: Front Oncol. 2025 Oct 14;15:1685758. doi: 10.3389/fonc.2025.1685758 (PMC12558770; doi:10.3389/fonc.2025.1685758)
Supplement: Supplementary file 2 [file DataSheet2.docx]

**Supplementary material**

**Supplementary Figures**

**Supplementary Figure Legends**

**Supplementary Figure 1 Penfluridol induces anti-tumor effects in human PCa cells**(A) Human PCa cell lines C4, C4-2 were exposed to a dose-range of penfluridol for 2 hours and subsequently the viability was assessed after 72 hours. Penfluridol induced a dose-dependent and significant reduction in viability in C4, C4-2 and C4-2B4 cells. Mean +/- standard error of the mean (SEM) * p<0.05, ** p<0.01, **** p<0.0001, one-way ANOVA. (B) Representative images of PC-3M-Pro4luc2, DU145, 22Rv1 and C4-2B4 cells exposed to penfluridol. Caspase-3/7 levels were increased after 24 hours upon penfluridol treatment. Treatment with 1 μM staurosporine was used as a positive control. Mean +/- standard error of the mean (SEM) * p<0.05, *** p<0.001, **** p<0.0001, One-way ANOVA. (C) Representative images of clonogenic assays of human PCa cell treated with penfluridol.

**Supplementary Figure 2 Penfluridol induces cancer cell death in PCa tissue slices**

Human PCa tumor tissue slices were *ex vivo* cultured and treated with penfluridol for 3 days. Cumulative scores of the four sections were calculated and displayed in heatmaps, with a higher score indicating a decrease in tissue quality. Scoring of PC-3M-Pro4luc2 CDX tissue slices (A) and PCa-15.01 and NM60 PDX tissue slices (B) revealed an increase in the cumulative score upon treatment with penfluridol, indicating an overall reduced tissue quality.

**Supplementary Figure 3 Synergistic effects of penfluridol in combination with docetaxel in docetaxel-resistant PCa cells**Docetaxel-sensitive PCa cells PC3, DU145 and 22Rv1 and docetaxel-resistant cells PC3-DR, DU145-DR and 22Rv1-DR were exposed to docetaxel, penfluridol or a combination of penfluridol and docetaxel. (A) Representative images of human PCa cells treated with docetaxel, penfluridol or a combination of docetaxel and penfluridol after 72 hours. The combination index was calculated and the standard error of the mean values were plotted in a table. (B-D) Viability of docetaxel-sensitive and docetaxel-resistant human PCa cells after treatment with docetaxel, penfluridol or a combination of both. The dotted line represents the expected combined effect of the sum of both monotherapies. In docetaxel-resistant PC3-DR, DU145-DR and 22Rv1-DR the observed effect of the combination therapy was stronger than the expected effect. Mean +/- standard error of the mean (SEM) * p<0.05, ** p<0.01, *** p<0.001, **** p<0.0001, One-way ANOVA

**Supplementary Tables**

**Supplementary Table 1 Cell culture media for two-dimensional cultures**

| **Cell line** | **Medium** | **Supplements** |
| --- | --- | --- |
| PC3 RRID:CVCV_0035 | Dulbecco’s Modified Eagle medium (DMEM) (Life technologies, Gibco, 31966-021) | 10% FCII (Hyclone), 100 units/ml penicillin, 50 μg/ml streptomycin (Life Technologies) |
| PC3-DR | Dulbecco’s Modified Eagle medium (DMEM) (Life technologies, Gibco, 31966-021) | 10% FCII (Hyclone), 100 units/ml penicillin, 50 μg/ml streptomycin (Life Technologies) + 12.5 nM docetaxel |
| PC-3M-Pro4luc2 (derived from PC-3M-Pro4 RRID:CVCL_D579) | Dulbecco’s Modified Eagle medium (DMEM) (Life technologies, Gibco, 31966-021) | 10% FCII (Hyclone), 100 units/ml penicillin, 50 μg/ml streptomycin (Life Technologies)  800 µg/mL of G-418 (Sigma) |
| DU145 (RRID:CVCL_0105) | RPMI 1640 (Lonza, BE12-167F) | 10% FBS, 100 units/ml penicillin,  50 μg/ml streptomycin (Life Technologies), GlutaMAX (Life Technologies) |
| DU145-DR | RPMI 1640 (Lonza, BE12-167F) | 10% FBS, 100 units/ml penicillin,  50 μg/ml streptomycin (Life Technologies), GlutaMAX (Life Technologies) + 12.5 nM docetaxel |
| 22Rv1  (RRID:CVCL_1045) | RPMI 1640 (Lonza, BE12-167F) | 10% FBS, 100 units/ml penicillin,  50 μg/ml streptomycin, GlutaMAX |
| 22Rv1-R (RRID:CVCL_4Y35) | RPMI 1640 (Lonza, BE12-167F) | 10% FBS, 100 units/ml penicillin,  50 μg/ml streptomycin, GlutaMAX + 12.5 nM docetaxel |
| C4-2B4  (RRID:CVCL_4787) | RPMI 1640 (Lonza, BE12-167F) | 10% FBS, 100 units/ml penicillin,  50 μg/ml streptomycin, GlutaMAX |

**Supplementary Table 2 Antibodies used for immunofluorescence stainings**

| **Target** | **Species** | **Supplier** | **Dilution** |
| --- | --- | --- | --- |
| Pancytokeratin | Rabbit | Abcam ab217916 | 1:500 |
| PCNA | Mouse | Sigma Aldrich P8825 | 1:2000 |
| Cleaved caspase-3 | Rabbit | Cell Signaling 9661L | 1:500 |
| Anti-mouse Alexa Fluor 488 | Donkey | Life Technologies A-21202 | 1:250 |
| Anti-rabbit Alexa Fluor 488 | Donkey | Life Technologies A-21206 | 1:250 |
| Anti-mouse Alexa Fluor 555 | Donkey | Life Technologies A-31570 | 1:250 |
| Anti-rabbit Alexa Fluor 555 | Donkey | Life Technologies A-31572 | 1:250 |

**Supplementary Table 3 Model and patient characteristics**

| **Tumour model / patient number** | **Source** | **Treatment history** | **Clinical status** | **References** |
| --- | --- | --- | --- | --- |
| MSK-PCa1 | L2 vertebral body | Androgen-deprivation therapy, bicalutamide | mCRPC | (20) |
| PCa-15.01 | Prostatectomy (hormone naïve) | No previous treatment | T3NxM+  Gleason 4+5  PSA>5000 ug/l | (21) |
| NM60 | Needle biopsy liver metastasis (CRPC) | Zoladex, Docetaxel, Abiraterone, radiotherapy, Cabazitaxel, Carboplatin, Olaparib | PSA: 81.0 ug/l | (21) |
| Patient #1 | Transurethral resection | EBRT, LHRH agonist, Enzalutamide | T4N+M+ Gleason 4+4  PSA: 52.5 ug/l | N.A. |
| Patient #2 | Transurethral resection | LHRH agonist | T3bNxMx  Gleason 3+4  PSA: 7.5 ug/l | N.A. |
| Patient #3 | Transurethral resection | Androgen deprivation | T3bN0Mx  Gleason 4+4  PSA: 15.0 ug/l | N.A. |
| Patient #4 | Transurethral resection | EBRT, Bicalutamide, Goserelin | T3aN0M0 Gleason 4+5  PSA: 14.23 ug/l | N.A. |
